# P114: Gombe Hospital hand hygiene project

**DOI:** 10.1186/2047-2994-2-S1-P114

**Published:** 2013-06-20

**Authors:** L Haruna

**Affiliations:** 1 Health, Gombe Hospital, Kampala, Uganda

## Introduction

Gombe Hospital is a 100-bed rural General public hospital situated 70 km west of Kampala, Butambala district in Uganda. Its catchment area is 300,000 people. The hospital staffing is 55% of the recommended. The hospital is facing a problem of inadequate water supply. This therefore made the observation of hand hygiene (HH) as recommended by WHO very difficult for both healthcare workers (HCW) and patients leading to high rates of healthcare-associated infections (HAI). Therefore a HH project was launched.

## Objectives

To strengthen hospital infection control in the context of inadequate water supply.

## Methods

Alcohol-based handrub (ABHR) were installed in wards and pocket size bottles were provided to HCWs, following training of all HCWs and how to use ABHR. HCWs were monitored for their compliance with the WHO “5 moments of HH” periodically and compliance rates calculated. The hospital also registered with WHO’s Clean Care Safer Care campaign as part of its commitment to Patient Safety.

## Results

HCWs’ compliance with the WHO five moments increased from 31% to 69% within 6 months of project implementation reflecting a positive change in attitude of HCWs towards HH. There was variation in compliance between departments and individual HCWs which was attributed to HCW attitudes, use of gloves, and time constraint. During a two weeks period when there was totally no water in the hospital operating theatre, ABHR was the only solution used for pre-operative scrubbing in 14 major operations and observed post-operative outcomes was the same as in the formal scrubbing when water and soap were used. Reduction in sepsis cases in maternity ward and in cross infection of diarrhea cases among the children in pediatrics ward was observed, as well as reduction in duration of hospital stay. ABHR was used also regularly by attendants and patients on the wards contributing to the noted reduction in ward sepsis.

## Conclusion

Compliance progressively improved over time which was an indication that HCWs owned the project and integrated the 5 WHO moments of HH concept. In Uganda and countries where health facility infrastructure is usually poor including no access to clean water, ABHR has big potential to improve hygiene condition without minimal expenditure on infrastructure renovation.

## Disclosure of interest

None declared

